# Evaluation of a Retrieval-Augmented Generation Chatbot for Antimicrobial Resistance Research: Comparative Analysis of Large Language Models

**DOI:** 10.2196/83206

**Published:** 2026-03-24

**Authors:** Oscar Escudero-Arnanz, Manuel Eduardo Valero-Méndez, Noelia Sánchez-Ramos, Cristina Soguero-Ruíz

**Affiliations:** 1 Department of Signal Theory and Communications, King Juan Carlos University, Camino del molino 5, Fuenlabrada, Madrid, 28943, Spain, 34689582328

**Keywords:** antimicrobial resistance, chatbot, large language models, retrieval-augmented generation, cost effectiveness evaluation, clinical decision support

## Abstract

**Background:**

Antimicrobial resistance (AMR) poses a critical global health threat, undermining the efficacy of antibiotics and complicating clinical decision-making. Although scientific literature on AMR is extensive, retrieving and synthesizing relevant evidence remains time-consuming for clinicians and researchers. Recent advances in large language models (LLMs) offer opportunities to enhance access to domain-specific knowledge. However, the diversity of available models, ranging from open-source to commercial, necessitates a systematic comparison of their performance, cost, and scalability in real-world biomedical applications.

**Objective:**

This study aims to describe the development of a retrieval-augmented generation (RAG) chatbot for AMR literature analysis and compare multiple commercial and open-source LLMs in terms of accuracy, faithfulness, response time, and cost-efficiency.

**Methods:**

A corpus of 164 peer-reviewed AMR-related articles was compiled from Google Scholar and embedded into a ChromaDB vector database using OpenAI’s text-embedding-ada-002. The RAG chatbot was implemented to operate with 5 LLM backbones: GPT-4, GPT-4o, GPT-4o-mini, Claude 3.7 Sonnet, and LLaMA 4 Maverick. For each model, a temperature ablation study was performed to determine optimal performance. Evaluation metrics included correctness (pass rate and score), faithfulness, relevancy, computational cost, and latency, using a synthetic ground truth dataset generated with GPT-4.

**Results:**

All models generated scientifically grounded responses when integrated into the RAG framework. GPT-4 achieved the highest correctness score (94.7%) but incurred the highest cost, while GPT-4o delivered nearly identical accuracy at a 9-fold lower cost and the fastest response time (3.88 s). LLaMA 4 Maverick and GPT-4o-mini offered lower accuracy but substantially reduced operational costs. Claude 3.7 Sonnet showed competitive accuracy, but the least favorable cost-performance ratio. Qualitative analysis revealed differences in response style, detail, and structure among models.

**Conclusions:**

A RAG-based chatbot can effectively support AMR research by delivering accurate, context-grounded, and scalable access to scientific literature. The comparative evaluation highlights trade-offs between performance, cost, and speed, guiding the selection of LLM architectures for clinical and research settings. Future work will focus on integrating language-specific embeddings and specialized domain agents to further enhance accuracy, adaptability, and clinical use.

## Introduction

Antimicrobial resistance (AMR), the capacity of microorganisms to withstand antimicrobial treatment, is one of the most urgent public health threats worldwide. It renders standard therapies ineffective, prolongs illness, increases health care costs, and raises mortality rates [1]. The World Health Organization estimates that, without effective interventions, AMR could cause up to 10 million deaths annually by 2050 [1]. In response, the scientific community has intensified research in areas such as the discovery of new antibiotics [2], identification of resistance mechanisms [3], and computational modeling for surveillance and prediction of resistance patterns [4].

However, the exponential growth of AMR-related literature has created a fragmented and complex knowledge landscape [5]. Researchers and clinicians face the challenge of rapidly locating, extracting, and synthesizing relevant findings, often under time constraints that make manual literature review inefficient or impractical.

Recent progress in large language models (LLMs) has opened new possibilities for improving biomedical knowledge retrieval and synthesis [6]. LLMs, trained on massive text corpora, can generate contextually relevant and coherent responses, making them suitable for biomedical question answering, health care administration, and clinical decision support [7-9]. Nevertheless, their performance varies widely depending on architecture, training data, and deployment model. Open-source models offer cost and customization advantages, while commercial solutions often provide higher baseline performance and stability. Selecting the most appropriate model involves balancing accuracy, speed, scalability, and budget constraints, factors particularly critical in health care environments.

One limitation of LLMs is their tendency to generate factually incorrect or fabricated information (“hallucinations”), which poses significant risks in clinical contexts [10]. Retrieval-augmented generation (RAG) mitigates this risk by grounding responses in dynamically retrieved, verified documents, improving factual accuracy and domain adaptability. Prior studies have demonstrated that RAG integration significantly reduces “hallucinations” and enhances reliability in medical reasoning [11]. While some RAG-based systems have been applied to public health education [12] or AMR policy development [13], few have targeted the efficient retrieval and synthesis of peer-reviewed AMR scientific literature.

This study aims to develop and evaluate a RAG-enabled chatbot designed to assist researchers and health care professionals in navigating AMR literature. Specifically, we sought to (1) compare the performance, cost, and response time of multiple commercial and open-source LLMs, and (2) provide an evidence-based cost-effectiveness evaluation framework to guide model selection for real-world biomedical applications.

## Methods

### Overview

**Figure 1.**
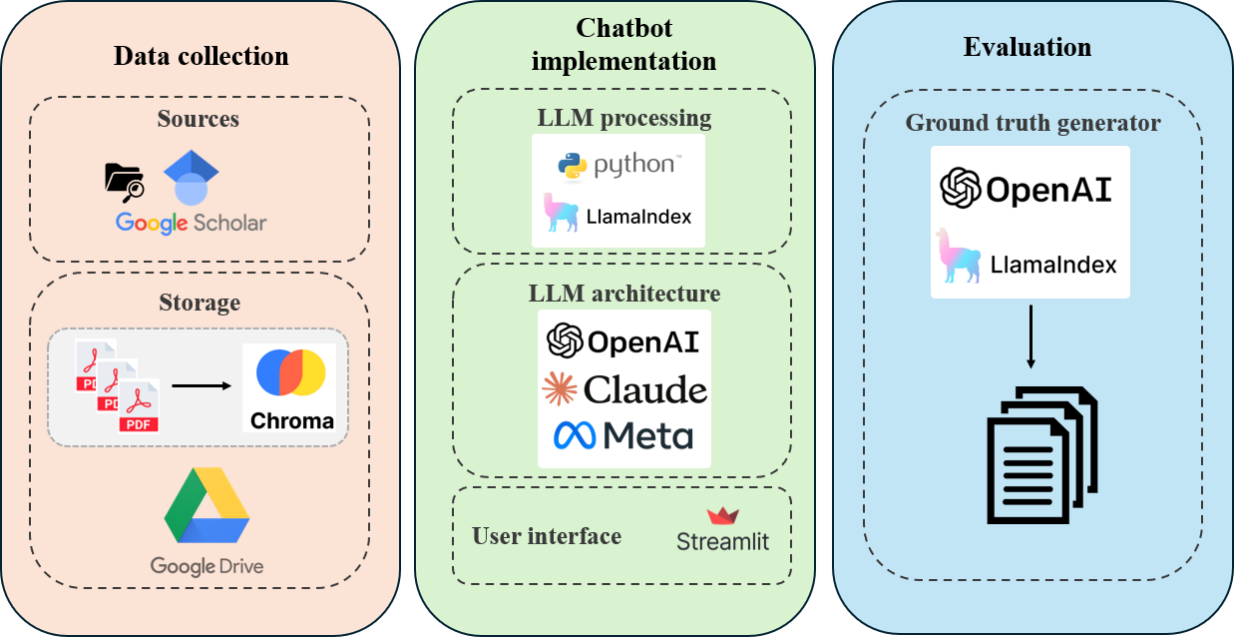
System architecture of the chatbot, illustrating document sources, storage layer, vector database, large language model processing stack, user-facing serving layer, and evaluation framework. LLM: large language model.

In this section, we present the system architecture (Figure 1) and its implementation, which follows a three-stage pipeline: (1) data collection, (2) chatbot deployment, and (3) evaluation methodology. The overarching goal is to enable efficient retrieval and generation of scientific information related to AMR through an interactive interface powered by generative artificial intelligence.

### Data Collection

The data collection process is a foundational component of this study, as it establishes the scientific knowledge base that powers the RAG chatbot architecture. Ensuring the relevance and reliability of the literature was essential to guarantee the quality of the chatbot’s output.

Relevant documents were manually retrieved using Google Scholar as the primary search engine, selected for its broad coverage of peer-reviewed literature and frequent updates, ensuring the inclusion of recent publications. The search strategy was guided by clinically relevant keywords related to AMR (Table 1), ensuring comprehensive topical coverage.

**Table 1.** Keywords used to search antimicrobial resistance-related scientific literature.

Antimicrobial resistance	Germs
Multidrug resistance	ESKAPE pathogens (*Enterococcus faecium*, *Staphylococcus aureus*, *Klebsiella pneumoniae*, *Acinetobacter baumannii*, *Pseudomonas aeruginosa*, and Enterobacter species)
Mechanical ventilation	WHO^a^ bacterial priority pathogens list
Microbiological culture	EHR^b^ and AMR^c^
Antibiotics	Machine learning for antimicrobial resistance
Microorganism	Deep learning for AMR prediction
Graph neural networks for AMR	EHRs and AMR
Interpretable AI^d^ AMR	AMR in intensive care unit
AMR clinical data	Risk factors for drug-resistant infections
Horizontal gene transfer AMR	Drug-resistant pathogens in hospitals
Beta-lactam resistance mechanisms	Efflux pumps and antibiotic resistance
Mutations and resistance evolution	Plasmid-mediated antimicrobial resistance
Carbapenem-resistant enterobacterales	Vancomycin-resistant enterococci
Methicillin-resistant *Staphylococcus aureus*	Extensively drug-resistant bacteria
Pan-drug-resistant bacteria	Efflux pumps and antibiotic resistance
Mutations and resistance evolution	ESBL^e^-producing bacteria

a WHO: World Health Organization.

b EHR: electronic health record.

c AMR: antimicrobial resistance.

d AI: artificial intelligence.

e ESBL: extended-spectrum beta-lactamases.

The retrieved documents were centrally stored in Google Drive in PDF format, which facilitated secure and structured management of the files for further processing.

The data preprocessing phase was conducted in close collaboration with clinicians from the Hospital Universitario de Fuenlabrada and under the expertise of the research group at Universidad Rey Juan Carlos, which has over 6 years of experience in the AMR domain. This expert-guided process ensured that each document was relevant to the study’s objectives and clinically meaningful. After applying these selection criteria, the final corpus comprised 164 clinically validated documents, including peer-reviewed scientific articles, technical reports, and specialized reviews covering diverse aspects of AMR.

To facilitate precise and efficient information retrieval, each document was segmented into smaller, semantically coherent units (chunks). This chunking strategy enables the system to retrieve the top *K* most relevant segments in response to a user query. The parameter *K* controls how many of the highest-ranked segments, based on semantic similarity, are retrieved for response generation. Each chunk was converted into a vector embedding using the text-embedding-ada-002 model from OpenAI [14], which encodes the semantic meaning of the text into a high-dimensional vector representation. These embeddings were stored in ChromaDB [15], a vector database optimized for semantic search using similarity metrics.

This hybrid approach, combining scientific literature retrieval (Google Scholar) and advanced semantic search (ChromaDB), provides a robust foundation for the chatbot system. This combination was selected for its efficiency, interoperability, and low operational cost, making it suitable for academic research settings and the functional prototyping of AI-driven literature exploration tools.

### Chatbot Implementation

The chatbot system was implemented on Microsoft Azure to ensure scalability and to facilitate a direct, controlled comparison of 5 state-of-the-art LLM architectures, each serving as the generative backbone in the experimental evaluation.

GPT-4 (OpenAI) [16] is a commercial Mixture-of-Experts (MoE) transformer model with approximately 1.8 trillion parameters. In an MoE architecture, several specialized submodels, known as experts, coexist alongside a gating network that, for each input, assigns weights and activates only the most relevant experts. The weighted outputs of those experts are then combined to produce the final output. It is accessible via a paid application programming interface and has demonstrated state-of-the-art performance across a wide range of benchmarks.

GPT-4o (OpenAI) [17] is a more efficient, multimodal variant of GPT-4. It delivers similar capabilities at lower latency and cost, with an extended context window, with approximately 200 billion parameters.

GPT-4o-mini (OpenAI) [18] is a compact version of GPT-4o, optimized for speed and computational efficiency with approximately 8 billion parameters.

Claude 3.7 Sonnet (Anthropic**)** [19] is a proprietary model fine-tuned for deep reasoning and following complex instructions, with approximately 140 billion parameters.

LLaMA 4 Maverick (Meta**)** [20] is an open-source, MoE model with 400 billion parameters overall, and it has a modular design that enables flexible deployment and customization in academic and industrial settings.

**Figure 2.**
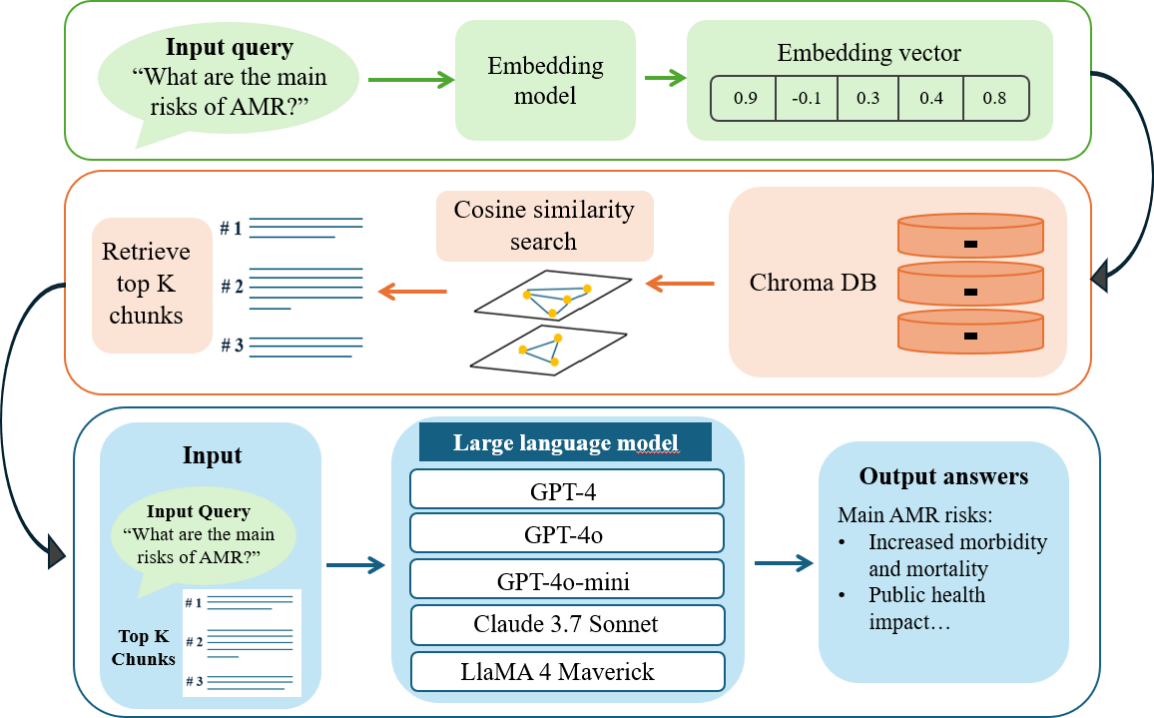
Schematic workflow of chatbot implementation. AMR: antimicrobial resistance.

The implementation workflow is illustrated in Figure 2. Upon receiving a user query, the system generates an embedding vector using OpenAI’s text-embedding-ada-002 model, ensuring compatibility with the vector representations used during document indexing. A semantic similarity search is then performed in ChromaDB, retrieving the top-3 most relevant document chunks based on cosine similarity. These retrieved chunks are concatenated with the user query to form the contextual input to the LLM, enabling the generation of responses that are both grounded in retrieved evidence and contextually coherent. This design ensures that each model is evaluated under identical retrieval conditions, thereby isolating differences in generative capabilities and cost-performance trade-offs.

The chatbot follows a RAG architecture to improve factual accuracy and contextual grounding. In this paradigm, generative reasoning is augmented with evidence-based retrieval, allowing the system to rely on verified documents rather than solely on the model’s internal parameters. This is particularly relevant in biomedicine, where precision, traceability, and reliability are paramount.

**Figure 3.**
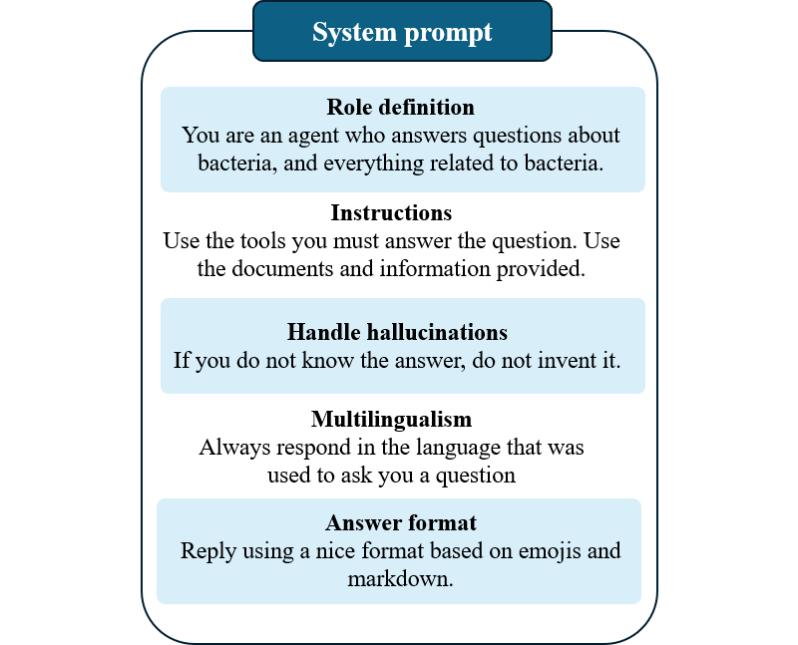
Illustration of prompt engineering to guide the behavior of the chatbot.

To guide response generation, we designed a specific system prompt (Figure 3) that instructed the model to rely strictly on retrieved context, avoid hallucinated or unsupported claims, respond using structured formatting, and adapt to the user’s language while incorporating emojis to enhance clarity and user engagement. This prompt was integrated into a custom agent tool, annotated with a domain-specific label indicating its suitability for addressing queries related to bacteria and antibiotics. This semantic labeling aimed to improve alignment during both the retrieval and generation phases.

The chatbot is deployed through a Streamlit web interface, providing a user-friendly environment for real-time interaction through a simple web user interface, making deployment and maintenance easier. There are 2 options for deploying the Streamlit interface: local deployment, which is useful during the development phase, and cloud deployment with Streamlit Cloud.

### Evaluation Methodology

The evaluation framework was designed to objectively assess the accuracy, relevance, and scientific grounding of the chatbot’s responses, given its focus on clinical scenarios and biomedical research contexts. Ensuring factual correctness and traceability is critical in these domains, where incorrect or unsupported information can have significant consequences.

A ground truth dataset was constructed from question–answering (QA) pairs derived directly from the scientific corpus indexed by the system. This approach enabled a direct, one-to-one comparison between the chatbot’s generated responses and validated reference answers. Ideally, such a dataset would be fully curated by domain experts; however, given the scope and volume of the indexed literature, manual creation of a complete benchmark was not feasible. Instead, we adopted a hybrid strategy, automatically generating the majority of QA pairs using GPT-4 [16], while incorporating a subset of expert-curated pairs to enrich the benchmark with domain-specific rigor.

For automated generation, GPT-4 was configured to produce consistent and deterministic outputs by setting the temperature parameter to 0.0. In LLMs, this parameter controls the degree of randomness in the output: lower values lead to more focused and repeatable responses, whereas higher values introduce greater variability. Setting the temperature to 0 ensured reproducibility across all generations.

**Figure 4.**
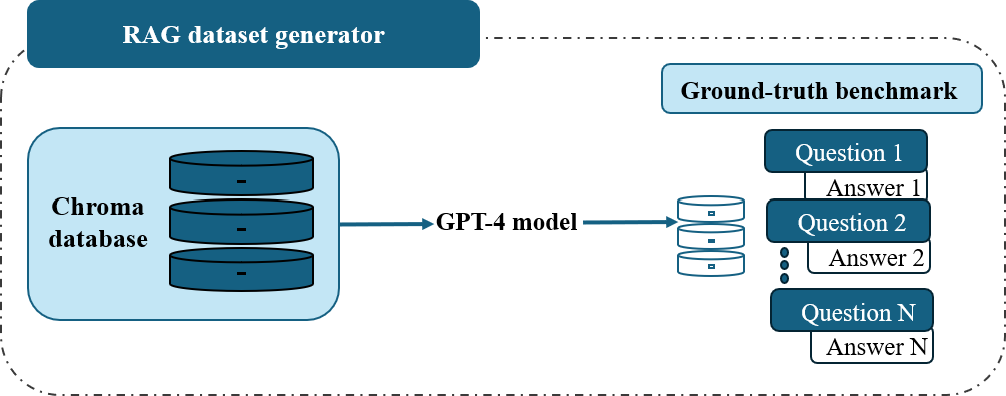
Overview of the automated ground-truth dataset generation process using the retrieval-augmented generation (RAG) Dataset generator.

The QA set generation process was implemented using the RagDatasetGenerator from LLaMAIndex [21], a framework specifically designed to support the creation of evaluation datasets for RAG systems (Figure 4). For each entry, the system generated a clinically or scientifically relevant question, a reference answer grounded in the retrieved evidence, and the supporting text fragment from the corpus used to derive that answer.

Once the benchmark dataset was created, the chatbot was prompted with the same set of questions, and its responses were systematically compared with the reference answers. This comparison assessed multiple dimensions, including content accuracy, defined as the degree of alignment with scientific evidence, contextual relevance, referring to the correct use of retrieved information, and factual consistency, which required the absence of unsupported claims or hallucinations [22].

This evaluation methodology provided a controlled and repeatable framework to measure the chatbot’s capacity to retrieve pertinent knowledge and generate reliable, context-aware outputs across different LLM backbones.

We implemented a multimetric evaluation to assess each chatbot’s outputs along 3 dimensions [23]:

1. Faithfulness evaluates whether the generated response is consistent with the retrieved context, without introducing hallucinated or unsupported information. The outcome is binary, indicating whether the response passes or fails this criterion.
2. Relevancy measures how directly and effectively the response addresses the user’s query. Like faithfulness, this metric returns a binary outcome: pass or fail.
3. Correctness assesses the degree of alignment between the chatbot’s answer and the predefined ground truth. This metric includes both:
  - Pass: whether the answer fulfills the correctness criterion (pass or fail).
  - Score: responses are rated on a Likert scale from 0 to 5 based on how well they align with the ground truth. These scores are normalized to percentages for comparative analysis.

To further clarify the evaluation framework, the “Correctness Pass” and “Correctness Score” in LlamaIndex assessments are closely related but capture different aspects of answer quality. The correctness pass is a binary indicator reflecting whether a response meets an acceptable correctness threshold. Evaluators assign Likert scale ratings (0‐5), and any response rated above a defined cutoff (typically 4 or 5) is considered a “pass,” while lower-rated responses are labeled as “fail.” This thresholding approach enables consistent interpretation and facilitates cross-model comparison by establishing a common criterion for what qualifies as sufficiently correct. Consequently, the correctness pass provides a straightforward percentage representing how often a model produces adequately correct outputs.

In contrast, the correctness score captures more nuanced distinctions by averaging the Likert ratings across all responses of the entire dataset. Each point on the 0‐5 Likert scale corresponds to a qualitative rubric ranging from “perfectly correct” (5) to “completely incorrect” (0), allowing evaluators to express varying degrees of alignment between generated and reference answers. This average score, often normalized to a percentage, reflects how close a model’s responses are, on average, to full correctness. Together, these 2 complementary measures provide a comprehensive view of performance, quantifying both the proportion of acceptable outputs and the overall degree of correctness, thereby enhancing the transparency and reproducibility of the evaluation process.

The evaluation framework was standardized across all models to ensure consistency and reproducibility. The exact prompts used to assess faithfulness, relevance, and correctness are provided in Multimedia Appendix 1.

### Ethical Considerations

Ethics board approval was not required for this study because it involved only the analysis of publicly available, anonymized data and did not include human participants or identifiable private information. In accordance with institutional and local research ethics policies, this type of research is exempt from Institutional Review Board review [24]. The study did not involve interaction with individuals, recruitment, or the collection of personal data; therefore, informed consent was not required. No compensation was provided, as no human participants were involved. All data analyzed were obtained from publicly accessible sources and processed in an aggregated, nonidentifiable form. No attempts were made to reidentify individuals, and no sensitive or private information was collected or reported. The study adhered to applicable data protection and research integrity standards.

## Results

**Figure 5.**
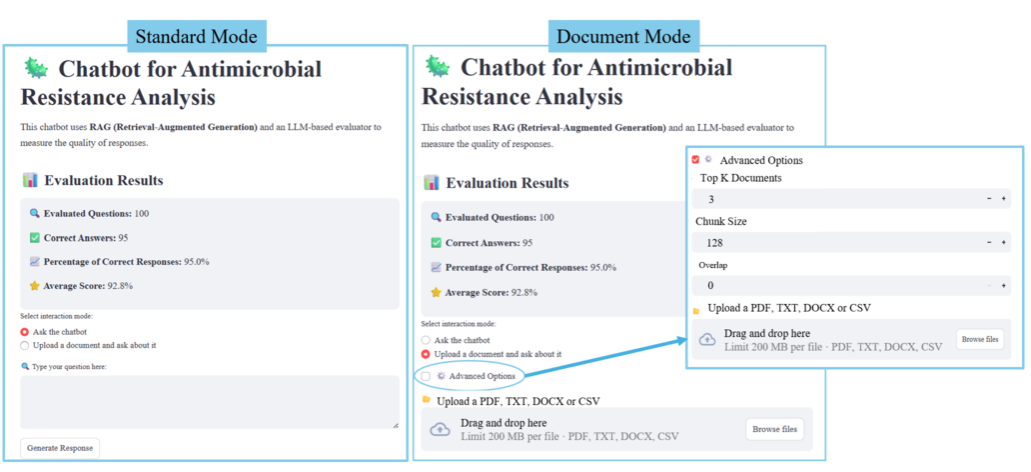
User interface for (A) standard mode and (B) document mode, where the user can upload a document and adjust the model parameters. RAG: retrieval-augmented generation.

The chatbot interface, presented in Figure 5, includes a clear title header, brief usage instructions, and a display of key evaluation metrics to provide users with transparency regarding the system’s performance. It supports 2 interaction modes. In the standard mode, users can submit free-text queries, and the chatbot automatically retrieves relevant information to generate a contextualized response. In the document mode, users are able to upload their own files and pose questions about their content; in this case, the system first indexes the uploaded document and then performs retrieval and generation in real time.

To enhance flexibility in document mode, an advanced settings panel allows users to fine-tune retrieval parameters, including *top K*, chunk size, and overlap. The chunk size defines the number of tokens per segmented passage, while the overlap specifies the number of tokens shared between consecutive chunks. Adjusting these parameters enables users to adapt the retrieval process to the structure of their documents and specific information needs, thereby improving accuracy and contextual relevance.

A series of controlled experiments was conducted to evaluate the performance of the proposed RAG-based chatbot across different LLM architectures. The evaluation was conducted using a predefined ground truth set of thirty representative questions related to AMR. The decision to use thirty test queries was both practical and methodologically sound, balancing computational efficiency with domain representativeness. Questions were automatically generated using LlamaIndex’s DatasetGenerator, which directly extracts content from the 164-article corpus to ensure comprehensive coverage across diverse AMR topics such as resistance mechanisms, clinical data, and predictive modeling. This tool supports stratified sampling, allowing questions to be evenly distributed by topic or difficulty, thereby minimizing sampling bias and enhancing the representativeness of the evaluation set relative to the full corpus.

The chosen sample size aligns with standard practices (“Notably, we constrain the number of QA pairs per document to a maximum of 20, with its range spanning from 1 to 16, aiming to better emulate real-world usage scenarios.” [25]) in domain-specific LLM benchmarking, offering a statistically meaningful yet computationally feasible foundation for cross-model comparison. This number provides sufficient variation to capture general performance trends without incurring excessive computational costs, making it suitable for academic research contexts with limited computing budgets. By combining stratified selection and automated generation, the evaluation design adheres to best practices in fairness, coverage, and methodological rigor.

Given the substantial impact of the temperature parameter on LLM output diversity and determinism, we systematically tested 4 values, 0.1, 0.3, 0.5, and 0.7, to assess their influence on response quality. The selected models were GPT-4o-mini, GPT-4, GPT-4o, Claude 3.7 Sonnet, and LLaMA 4 Maverick. System performance was evaluated using 3 previously defined metrics: faithfulness, relevancy, and correctness. For each model-temperature configuration, we recorded the proportion of responses passing each metric, the average correctness score (normalized to a percentage), and the average response time (in seconds) to account for computational efficiency and user experience.

**Figure 6.**
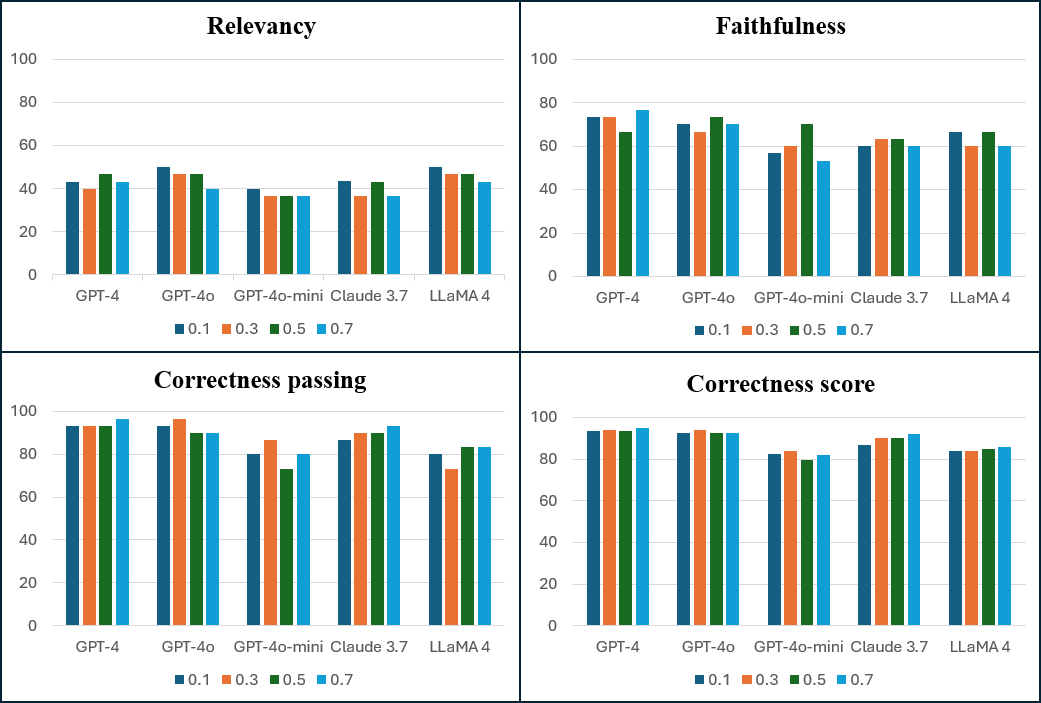
Performance comparison of large language models across four temperature settings on different evaluation metrics: relevancy, faithfulness, correctness passing, and correctness score.

Table 2 summarizes the performance of each model across all temperature settings, while Figure 6 provides a visual comparison. Relevancy scores generally ranged from 40% to 50% across all configurations, with only modest variations and no consistent trend suggesting systematic improvement or decline as temperature increased. Faithfulness exhibited higher overall performance, typically between 60% and 80%, with the highest value achieved by GPT-4 at temperature 0.7 (≈77%). GPT-4o-mini displayed the greatest variability in this metric, peaking at 70% at temperature 0.5 but dropping sharply to 53.3% at 0.7, the steepest decline among all models. Correctness consistently yielded the highest scores, ranging from 80% to 95%. In most cases, peak correctness was observed at temperature 0.3 for GPT-4o and GPT-4o-mini, and at 0.7 for GPT-4, Claude 3.7, and LLaMA 4.

**Table 2.** Evaluation results for different temperature configurations.

Models and temperatures		Relevancy (%)	Faithfulness (%)	Correctness passing (%)	Correctness score (%)
GPT-4					
0.1		43.3	73.3	93.3	93.3
0.3		40	73.3	93.3	94
0.5		46.7	66.7	93.3	93.3
0.7		43.3	76.7	96.7	94.7
GPT-4o					
0.1		50	70	93.3	92.7
0.3		46.7	66.7	96.7	94
0.5		46.7	73.3	90	92.7
0.7		40	70	90	92.7
GPT-4o-mini					
0.1		40	56.7	80	82.3
0.3		36.7	60	86.7	84
0.5		36.7	70	73.3	79.7
0.7		36.7	53.3	80	82
Claude 3.7 Sonnet					
0.1		43.4	60	86.7	86.7
0.3		36.7	63.3	90	90
0.5		43.3	63.3	90	90
0.7		36.7	60	93.3	92
LLaMA 4 Maverick					
0.1		50	66.7	80	83.7
0.3		46.7	60	73.3	84
0.5		46.7	66.7	83.3	84.7
0.7		43.3	60	83.3	86

To complement the quantitative analysis, we qualitatively assessed the content and structure of model outputs using 3 domain-specific questions of increasing difficulty: easy, intermediate, and difficult. Each model was tested using the temperature setting that yielded its highest correctness score—0.3 for GPT-4o and GPT-4o-mini, and 0.7 for GPT-4, Claude 3.7, and LLaMA 4.

For the easy-level question (Textbox 1)**,** the larger, high-capacity models (GPT-4, GPT-4o, and Claude 3.7) consistently produced more elaborate and well-structured responses, often organized into thematic categories with rich contextual details. In contrast, GPT-4o-mini and LLaMA 4, which are either more compact or open-source models, tended to generate shorter, more generic answers with less contextual depth.

Textbox 1. **Model responses to the question: “Which germs are known for their multidrug-resistant (MDR) potential?”**

**GPT-4**

- Various germs are known for their multidrug-resistant (MDR) potential. Among the most clinically significant are (1) Bacteria: *Pseudomonas aeruginosa* (resistant to multiple antibiotics and capable of forming biofilms); Enterobacterales, including carbapenem-resistant strains; *Klebsiella pneumoniae*, especially pan–drug-resistant (PDR) strains; *Mycobacterium tuberculosis* (MDR-TB); (2) Fungi: *Candida auris* (high antifungal resistance); *Aspergillus fumigatus* (azole resistance); (3) Viruses: Influenza A (H1N1) and HIV, which have developed resistance to antivirals. These organisms pose major challenges to treatment and global health.

**GPT-4o**

- The germs known for their MDR potential include (1) Bacteria: *Pseudomonas aeruginosa*; mdr Enterobacterales; carbapenem-resistant *Klebsiella pneumoniae*; *Mycobacterium tuberculosis* (MDR-TB); (2) Fungi: *Candida auris*; *Aspergillus fumigatus*; (3) Viruses: Influenza A (H1N1) and HIV with antiviral resistance. These microorganisms complicate treatment due to resistance to multiple therapeutic options.

**Claude 3.7**

- Several microorganisms are known for MDR potential, including (1) Bacteria: *Pseudomonas aeruginosa* and carbapenem-resistant Enterobacterales; *Klebsiella pneumoniae* (PDR); *Mycobacterium tuberculosis* (MDR-TB); (2) Fungi: *Candida auris* and *Aspergillus fumigatus* show antifungal resistance; (3) Viruses, including HIV and Influenza A (H1N1), exhibit antiviral resistance. MDR Gram-negative bacteria have increased in prevalence in recent years.

**GPT-4o-mini**

- The germs known for MDR include *Pseudomonas aeruginosa*, *Klebsiella pneumoniae*, and *Mycobacterium tuberculosis*. *Pseudomonas aeruginosa* is significant in cystic fibrosis infections; *Klebsiella pneumoniae* PDR strains can cause severe bacteremia; MDR-TB remains a global threat.

**LLaMA 4**

- Several germs with MDR potential include (1) Bacteria: *Pseudomonas aeruginosa*, Enterobacterales, *Klebsiella pneumoniae*, and *Mycobacterium tuberculosis*; (2) Fungi such as *Candida auris* and *Aspergillus fumigatus*; and (3) Viruses: HIV and Influenza A (H1N1). These pathogens have developed resistance to multiple drugs, complicating treatment.

For the intermediate-level question (Textbox 2), GPT-4 and GPT-4o produced less structured outputs than in the easy-level scenario, while Claude 3.7 adopted a more enumerated and organized presentation. GPT-4o-mini and LLaMA 4 again delivered simpler, less specific responses, showing a notable similarity in style and depth. Despite the reduced structural organization, GPT-4 and GPT-4o maintained stylistic consistency across tasks.

Textbox 2. **Model responses to the intermediate-level question: “What are the main risk factors for developing drug-resistant ventilator-associated pneumonia (VAP) in adult patients?”**

**GPT-4**

- The main risk factors for developing drug-resistant ventilator-associated pneumonia (VAP) in adult patients include prolonged use of mechanical ventilation, prior use of broad-spectrum antibiotics such as carbapenems, the presence of severe conditions like severe sepsis or septic shock, and the involvement of multidrug-resistant (MDR) pathogens such as *Acinetobacter baumannii* and *Pseudomonas aeruginosa*. Additionally, factors such as low Glasgow Coma Scale scores due to trauma or severe neurological conditions can increase susceptibility to infections, as can the lack of proper antibiotic stewardship policies, which contributes to a high prevalence of multidrug-resistant bacteria.

**GPT-4o**

- The main risk factors for developing drug-resistant VAP in adult patients include prolonged use of mechanical ventilation, prior administration of broad-spectrum antibiotics such as carbapenems, the presence of severe comorbidities, such as subdural hematomas or brain injuries, that impair immunity, and the onset of late-onset VAP. In addition, the lack of strict antibiotic use policies and the misuse of empirical therapies significantly contribute to the development of MDR organisms such as *Acinetobacter baumannii*, *Pseudomonas aeruginosa*, and methicillin-resistant *Staphylococcus aureus* (MRSA).

**Claude 3.7**

- Based on the information provided, the main risk factors for developing drug-resistant VAP in adult patients include:

- Prolonged use of mechanical ventilation (the reported mean duration was 10-10.5 days)
- Late-onset pneumonia
- Septic shock and severe sepsis
- Multilobar pneumonia
- Bilateral lung involvement
- Prior use of carbapenems within the 72 hours preceding pneumonia onset
- Infection caused by specific organisms such as MDR *Acinetobacter baumannii* (PDR) or *Pseudomonas aeruginosa*
- Low Glasgow Coma Scale scores (associated with subdural hematoma and status epilepticus)
- The study also highlights that the lack of proper antibiotic policies and the inappropriate use of empirical antibiotics significantly contribute to high rates of MDR in hospital settings.

**GPT-4o-mini**

- The main risk factors for developing drug-resistant VAP in adult patients include late-onset pneumonia, septic shock, severe sepsis, multilobar pneumonia, bilateral lung involvement, and prior use of carbapenems within the 72 hours preceding the onset of pneumonia. Additionally, the presence of MDR organisms such as *Acinetobacter baumannii* (PDR) and *Pseudomonas aeruginosa* is also associated with worse clinical outcomes.

**LLaMA 4**

- The main risk factors for developing drug-resistant VAP in adult patients include prior use of carbapenems within the 72 hours preceding the onset of pneumonia, the presence of septic shock, bilateral lung involvement, and pneumonia caused by pandrug-resistant bacteria such as *Acinetobacter baumannii* (PDR) or *Pseudomonas aeruginosa*. Additionally, prolonged mechanical ventilation and the severity of the patient's underlying condition, as reflected by a low Glasgow Coma Scale score, may also increase the risk of developing drug-resistant VAP.

In the difficult-level question (Textbox 3), GPT-4o-mini and LLaMA 4 produced outputs more closely resembling those of the larger models, suggesting improved performance under more challenging input. The three OpenAI models (GPT-4, GPT-4o, and GPT-4o-mini) presented standardized formatting, whereas Claude 3.7 retained its characteristic enumerated structure. Interestingly, LLaMA 4 diverged from this pattern, opting for an unnumbered format that distinguished its style from the rest.

Textbox 3. **Model responses to the difficult-level question: “Why do ESKAPE group pathogens represent a major concern in the context of antimicrobial resistance, and which pathogens are included in this group?”**

**GPT-4**

- ESKAPE group pathogens represent a major concern in the context of antimicrobial resistance due to their ability to evade the biocidal action of antimicrobial agents, making them a significant cause of severe nosocomial infections, particularly in critically ill and immunocompromised patients. These infections are often associated with worse clinical outcomes, such as prolonged hospital stays, higher mortality rates, and increased hospitalization costs. Moreover, the antimicrobial resistance of these pathogens contributes to the ineffectiveness of conventional treatments, complicating their management.
- The ESKAPE group includes six major pathogens responsible for most drug-resistant nosocomial infections:

- *Enterococcus faecium*
- *Staphylococcus aureus*
- *Klebsiella pneumoniae*
- *Acinetobacter baumannii*
- *Pseudomonas aeruginosa*
- *Enterobacter spp.*

- These organisms are a global concern due to their increasing resistance and their impact on public health and health care systems.

**GPT-4o**

- ESKAPE group pathogens represent a major concern in the context of antimicrobial resistance because they are responsible for the most severe nosocomial infections and have the ability to evade the biocidal action of antimicrobial agents. This makes them a significant threat, especially for critically ill and immunocompromised patients, as infections caused by resistant strains are associated with worse clinical outcomes—such as longer hospital stays, higher mortality rates, and increased hospitalization costs. Furthermore, their multidrug resistance (MDR) hinders effective treatment, worsening the issue.
- The ESKAPE pathogens include the following bacteria:

- *Enterococcus faecium*
- *Staphylococcus aureus*
- *Klebsiella pneumoniae*
- *Acinetobacter baumannii*
- *Pseudomonas aeruginosa*
- *Enterobacter spp.*

- These microorganisms are particularly problematic in hospital settings due to their ability to develop and spread resistance to multiple classes of antibiotics.

**Claude 3.7**

- ESKAPE group pathogens represent a major concern in the context of antimicrobial resistance because:

- They are responsible for approximately two-thirds of all health care–associated infections
- They contribute significantly to global morbidity and mortality, especially in intensive care units
- They exhibit both virulence and MDR
- They can “escape” the biocidal action of antimicrobial agents
- Infections caused by resistant strains of these pathogens are associated with worse clinical outcomes, including longer hospital stays, higher mortality and increased hospitalization costs

- The pathogens that make up the ESKAPE group are:

- E: *Enterococcus faecium*
- S: *Staphylococcus aureus*
- K: *Klebsiella pneumoniae*
- A: *Acinetobacter baumannii*
- P: *Pseudomonas aeruginosa*
- E: *Enterobacter species*

- These organisms are common causes of life-threatening infections worldwide, particularly in critically ill and immunocompromised patients.

**GPT-4o-mini**

- ESKAPE group pathogens represent a major concern in the context of antimicrobial resistance due to their ability to evade the action of antimicrobial agents and their association with severe nosocomial infections. These organisms are responsible for a large proportion of hospital-acquired infections and are linked to high morbidity and mortality rates, especially in immunocompromised patients. Their multidrug resistance makes treatment more difficult and contributes to poorer clinical outcomes, such as prolonged hospital stays and higher health care costs.
- The pathogens that make up the ESKAPE group are:

- *Enterococcus faecium*
- *Staphylococcus aureus*
- *Klebsiella pneumoniae*
- *Acinetobacter baumannii*
- *Pseudomonas aeruginosa*
- *Enterobacter spp.*

- These microorganisms are known for their virulence and resistance to multiple drugs, making them a significant challenge for infection control in health care settings.

**LLaMA 4**

- ESKAPE group pathogens represent a significant concern in the context of antimicrobial resistance due to their ability to cause nosocomial infections and their tendency to develop resistance to multiple antimicrobial drugs. These pathogens include *Enterococcus faecium*, *Staphylococcus aureus*, *Klebsiella pneumoniae*, *Acinetobacter baumannii*, *Pseudomonas aeruginosa*, and Enterobacter spp. Antimicrobial resistance in these pathogens is due to several mechanisms, including the production of enzymes that inactivate antibiotics, such as beta-lactamases, and modification of antibiotic targets. The spread of these resistant pathogens in health care settings can lead to increased morbidity, mortality, and healthcare costs. ESKAPE pathogens are responsible for a significant proportion of health care–associated infections and continue to play a major role in global mortality, especially in intensive care units.

**Figure 7.**
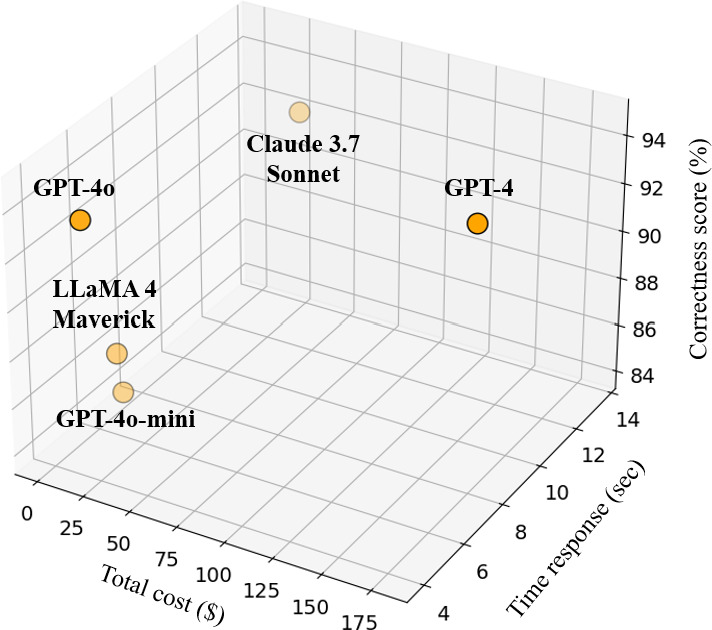
Relationship between correctness score, total cost per million tokens and time response for the evaluated large language models.

Table 3 presents the average response time for each model under its best-performing temperature setting, along with input and output costs. Pricing for Claude 3.7 [26] and LLaMA 4 [27] was obtained from Azure Databricks, and for GPT models from Azure OpenAI [28], all deployed in the Sweden Central region (note that prices may vary depending on deployment region or provider configurations). Figure 7 visualizes the trade-off between correctness scores, total cost, and response time.

**Table 3.** Time response and costs per million tokens.

Models	GPT-4	GPT-4o	GPT-4o-mini	Claude 3.7 Sonnet	LLaMA 4 Maverick
Time (seconds)	7.68	3.88	7.06	13.53	6.7
Input (in US $)	120	5	0.17	4.5	0.59
Output (in US $)	60	15	0.66	22.5	1.76

The results indicate that GPT-4, GPT-4o-mini, and LLaMA 4 Maverick achieved similar latencies, averaging around 7 seconds. GPT-4o was the fastest model, with an average response time of 3.88 seconds, offering a substantial efficiency advantage. Conversely, Claude 3.7 Sonnet exhibited the slowest performance, with an average of 13.53 seconds, over 3 times slower than GPT-4o.

In terms of cost-effectiveness, GPT-4 achieved the highest correctness score (94.7%) but at a cost of US $180 (input and output cost) per million tokens, making it the most expensive option. GPT-4o achieved a nearly identical score (94%) at a cost almost 9 times lower, clearly positioning it as the most cost-efficient high-performance choice. Claude 3.7 Sonnet attained a slightly lower correctness score (92%) while being more expensive, resulting in a less favorable cost–performance ratio. LLaMA 4 Maverick and GPT-4o-mini formed a lower-cost segment with moderate performance, reaching 86% (n/N) and 84% (n/N), respectively. Although GPT-4o-mini was marginally cheaper ($0.83 vs $2.35 per million tokens), LLaMA 4, as an open-source model, offered a compelling cost–accuracy balance, highlighting its potential as a competitive option in budget-constrained scenarios.

## Discussion

### Effects of Temperature Ablation

Our findings confirm the effectiveness of the proposed RAG-based chatbot in supporting AMR research by generating scientifically grounded, contextually relevant responses. This demonstrates its potential to streamline access to specialized knowledge in a reliable and scalable manner, particularly in scenarios where manual literature review is both time-consuming and resource-intensive.

The quality of the generated responses remained largely consistent across the evaluated temperature settings, with no single configuration universally outperforming the others across all models. This suggests that the choice of temperature should be guided by the intended use case, prioritizing rigor and precision for research-focused applications or fostering creativity and exploration for more exploratory or hypothesis-generating tasks. In parallel, our findings on response time reveal meaningful differences in computational efficiency, underscoring that model selection should also account for the time constraints of the target application, especially in time-critical environments.

### Model Selection and Cost-Time Performance Trade-Offs

The comparative evaluation of LLMs revealed important trade-offs between performance, cost, and latency. Among commercial offerings, GPT-4o emerged as the most balanced option, achieving high accuracy scores at a moderate cost while delivering the fastest average response time. This combination makes it well-suited for applications that demand high-quality outputs and efficient execution.

Claude 3.7 Sonnet also achieved strong results; however, its slightly lower accuracy, higher cost, and significantly longer response times, over 3 times slower than GPT-4o, reduce its cost-effectiveness, particularly in real-time or large-scale deployments. In contrast, LLaMA 4 Maverick, the only open-source model in the comparison, offered lower correctness scores but exceptionally low deployment costs. This affordability, combined with acceptable performance and moderate latency, positions it as a competitive alternative for organizations with limited resources or for large-scale implementations where cost efficiency is a priority.

GPT-4o-mini, although the least expensive, ranked lowest in accuracy and, as a proprietary model, lacks the flexibility and transparency of open-source solutions like LLaMA 4. This could limit its adoption in settings where model customization, auditing, or full control over the deployment pipeline is required.

From a qualitative perspective, model outputs differed noticeably in style, structure, and depth. Some LLMs generated highly structured and enumerated answers, while others favored more concise, narrative responses. This variability suggests that model selection should also consider the desired user experience, enabling alignment between output style and the end user’s expectations or the communication requirements of the target application.

### Advantages of RAG Architecture

The integration of a RAG framework enables the chatbot to overcome the inherent limitations of purely generative LLMs, which rely exclusively on static, pretrained internal knowledge. By incorporating an external retrieval step, the system dynamically grounds responses in relevant, up-to-date sources, ensuring factual alignment with current scientific evidence.

This design not only improves contextual accuracy and reduces the risk of hallucinations but also enhances the adaptability of the chatbot to rapidly evolving fields such as AMR research, where new findings and guidelines are continuously emerging. As a result, the chatbot delivers responses that are linguistically coherent, scientifically valid, and verifiable, attributes that are essential for supporting informed decision-making in clinical and research settings.

### Limitations

Despite the strengths of the proposed approach, several limitations should be acknowledged.

First, the embedding model used in this study, OpenAI’s text-embedding-ada-002, was primarily trained on large-scale general English-language datasets such as web text, Wikipedia, and open-domain corpora. This broad linguistic exposure enables robust semantic representations across diverse topics and ensures strong baseline performance for English-language queries, making it a practical and resource-efficient option for a mixed or exploratory RAG setup. However, because text-embedding-ada-002 is not specifically tuned on biomedical literature (eg, PubMed abstracts or clinical reports), it may underperform in capturing fine-grained domain-specific relationships, such as molecular interactions, specialized clinical terminology, or context-dependent abbreviations common in scientific writing. Although its general-purpose nature likely provided sufficient coverage for the goals of this evaluation, future research could compare domain-specialized embedding models, particularly those trained on biomedical corpora, to assess potential improvements in retrieval precision and overall RAG performance.

Another limitation arises from the use of LLMs for both data generation and evaluation. Because the synthetic question–answer pairs were produced by GPT-4 and the same class of models was subsequently used to generate and assess responses, some degree of circularity or model bias cannot be fully excluded. Although this design ensured consistency and scalability in benchmarking, it may favor models that share similar linguistic or reasoning patterns with the generator. To mitigate this risk, evaluation prompts were standardized across all models, and metrics were computed independently for each output. Future work should incorporate complementary validation strategies, such as expert-driven assessment or cross-model judging, to enhance objectivity and robustness.

Second, response latency can increase notably when processing large documents or handling real-time uploads, potentially impacting user experience in high-demand scenarios.

Finally, the chatbot currently operates as a single generalist agent covering the entire AMR knowledge base, which may constrain its ability to provide highly specialized reasoning within subdomains. Future work should investigate modular or agent-specialized designs to improve domain-specific precision and adaptability.

### Conclusions

This study demonstrates the feasibility and effectiveness of a RAG-based chatbot to support scientific research on AMR by combining LLM generation with dynamic retrieval from domain-specific literature. The proposed architecture is robust, modular, and scalable, enabling flexible adaptation to diverse operational settings. The findings highlight a clear trade-off between accuracy, cost, and response time, with open-source models such as LLaMA 4 offering a viable, cost-effective solution for large-scale or resource-constrained environments, and premium models such as GPT-4o delivering superior accuracy and efficiency for high-stakes applications. Beyond its immediate use in AMR, the approach presented here can be generalized to other biomedical domains requiring rapid, evidence-grounded information access. Future research should explore the integration of language-specific embeddings, fine-tuning with domain corpora, and the deployment of specialized domain agents to enhance contextual precision. Additionally, efforts will focus on creating more conversational, adaptive, and explainable interactions to foster user engagement and trust, thereby positioning this system as a reliable, long-term tool for evidence-based decision-making.

## Supplementary material

10.2196/83206 Multimedia Appendix 1 Evaluation prompts for faithfulness, relevancy, and correctness.

## Abbreviations

AMR: antimicrobial resistance
LLM: large language model
MoE: Mixture-of-Experts
QA: question–answering
RAG: retrieval-augmented generation
