# Supplementary material for: Evaluation of a Retrieval-Augmented Generation Chatbot for Antimicrobial Resistance Research: Comparative Analysis of Large Language Models
Source: JMIR AI. 2026 Mar 24;5:e83206. doi: 10.2196/83206 (PMC13012410; doi:10.2196/83206)
Supplement: Multimedia Appendix 1 [file ai-v5-e83206-s001.docx]

# Evaluation Prompts for Faithfulness, Relevancy, and Correctness

The following templates show the exact evaluation prompts used across all models to assess *correctness, relevancy and faithfulness*. These prompts were implemented within the LlamaIndex evaluation pipeline and remained identical for all models to ensure a fair and consistent comparison.

# Correctness

## Default system template

You are an expert evaluation system for a question-answering chatbot.

You are given the following information:

- A user query, and
- A generated answer.

You may also be given a reference answer to use for comparison in your evaluation.

Your job is to judge the relevance and correctness of the generated answer.

Output a single score that represents a holistic evaluation.

You must return your response in line with only the score. Do not return answers in any other format.

On a separate line, provide your reasoning for the score as well.

Follow these guidelines for scoring:

- Your score must be between 1 and 5, where 1 is the worst and 5 is the best.
- If the generated answer is not relevant to the user query, give a score of 1.
- If the generated answer is relevant but contains mistakes, give a score between 2 and 3.
- If the generated answer is relevant and fully correct, give a score between 4 and 5.

## Example response

4.0

The generated answer has the exact same metrics as the reference answer, but it is not as concise.

## Default user template

{query}

## Reference Answer

{reference_answer}

## Generated Answer

{generated_answer}

# Relevance

## Default evaluation template

Your task is to evaluate whether the response to the query is in line with the context information provided. You have two options to answer: either YES or NO.

Answer YES if the response to the query is in line with the context information; otherwise, answer NO.

Query and Response:

{query_str}

Context:

{context_str}

Answer:

## Default refinement template

We want to understand if the following query and response are in line with the context information:

{query_str}

We have provided an existing YES/NO answer:

{existing_answer}

We have the opportunity to refine the existing answer (only if needed) with some more context below:

{context_msg}

If the existing answer was already YES, still answer YES.

If the information is present in the new context, answer YES.

Otherwise, answer NO.

# Faithfulness

## Default evaluation template

Please tell if a given piece of information is supported by the context.

You must answer with either YES or NO.

Answer YES if any part of the context supports the information, even if most of the context is unrelated.

## Examples

Information: Apple pie is generally double-crusted.

Context: An apple pie is a fruit pie in which the principal filling ingredient is apples. Apple pie is often served with whipped cream, ice cream, custard, or cheddar cheese. It is generally double-crusted, with pastry both above and below the filling; the upper crust may be solid or latticed (woven of crosswise strips).

Answer: YES

Information: Apple pies taste bad.

Context: An apple pie is a fruit pie in which the principal filling ingredient is apples. Apple pie is often served with whipped cream, ice cream, custard, or cheddar cheese. It is generally double-crusted, with pastry both above and below the filling; the upper crust may be solid or latticed (woven of crosswise strips).

Answer: NO

## Default evaluation format

Information: {query_str}

Context: {context_str}

Answer:

## Default refinement template

We want to understand if the following information is present in the context information:

{query_str}

We have provided an existing YES/NO answer:

{existing_answer}

We have the opportunity to refine the existing answer (only if needed) with some more context below:

{context_msg}

If the existing answer was already YES, still answer YES.

If the information is present in the new context, answer YES.

Otherwise, answer NO.
